# COVID-19 vaccine hesitancy prevalence in Mexico: A systematic review and metanalysis

**DOI:** 10.1016/j.jvacx.2024.100488

**Published:** 2024-04-21

**Authors:** Diego Ramonfaur, Rupali J. Limaye, David E. Hinojosa-González, Francisco J. Barrera, Gloria P. Rodríguez-Gómez, Carlos Castillo-Salgado

**Affiliations:** a Bloomberg School of Public Health, Johns Hopkins University, Baltimore, MD, USA; b Cleveland Clinic, Department of Internal Medicine, Cleveland, OH, USA; c Department of International Health, Johns Hopkins Bloomberg School of Public Health, Baltimore, MD, USA; d International Vaccine Access Center, Johns Hopkins Bloomberg School of Public Health, Baltimore, MD, USA; e Baylor College of Medicine, Houston, TX, USA; f Department of Psychiatry, Massachusetts General Hospital, Harvard Medical School, Boston, MA, USA; g Yale New Haven Hospital, Yale School of Medicine, New Haven, CT, USA

**Keywords:** COVID-19, Vaccine hesitancy, Public Health, Epidemiology, Latin America

## Abstract

**Background:**

Vaccine hesitancy (VH) is a recognized threat to public health that undermines efforts to mitigate disease burden. This study aims to gather available evidence regarding COVID-19 VH in Mexico, estimate the prevalence of VH, and its determinants to inform policymaking in this country.

**Methods:**

Following PRISMA guidelines, a systematic review of the MEDLINE literature, articles that estimated the prevalence of COVID-19 VH in Mexico were included in the analysis to obtain a pooled estimate. We used a binomial-normal model for *meta*-analysis of proportions (i.e., generalized linear mixed model) to perform the metanalysis. We then performed a narrative review of COVID-19 VH in Mexican subpopulations.

**Results:**

Seven studies met inclusion criteria. We estimated a pooled prevalence of COVID-19 VH of 16 % (95 % CI: 11–23 %) in Mexico. We found an association between VH and demographic characteristics, intrinsic vaccine factors, and beliefs. Subgroup analyses from specific studies suggested that patients with clinical conditions such as breast cancer or rheumatologic diseases had a higher prevalence of VH.

**Conclusions:**

VH is a highly complex and dynamic phenomenon in Mexico. Characterizing and understanding COVID-19 vaccine hesitancy in the Mexican population helps target future policy interventions to mitigate the spread and impact of infectious diseases. The implications of VH differ among groups that may be at higher risk of severe disease, underscoring the importance of prompt research among these groups as well as targeted interventions to address VH.

## Introduction

Coronavirus disease 2019 (COVID-19) is a vaccine-preventable viral infectious disease[1] that continues to pose a massive challenge to healthcare systems worldwide, leading to considerable morbidity and mortality. According to the most recent available estimates, COVID-19 is responsible for approximately 7 million deaths worldwide, with at least 350,000 deaths in Mexico attributed to COVID-19.[2] Incidence and mortality estimates are likely on the conservative side, underestimating the burden and effects of the disease.[3] Vaccine research groups successfully trialed dozens of vaccine candidates that have proven safe and effective in preventing symptomatic COVID-19 disease, disease progression, and death,[4] even in the context of novel and more virulent variants.[5] While several studies have examined the safety and effectiveness of COVID-19 vaccines,[6], [7], [8], [9] a considerable proportion of individuals worldwide remain unwilling to be immunized.[10], [11], [12].

Vaccine hesitancy (VH) – defined as the delay in acceptance or refusal of vaccination despite the availability of the vaccination services – is a complex and multifaceted issue that can be understood through three lenses: contextual, individual, and vaccine-specific.[13] VH is highly variable across regions[11], [12]and age strata[14], [15] as well as by disease.[16], [17] The prevalence of COVID-19 VH in Mexico has been estimated to be between 10 and 40 %[11], [15], [18], [19], [20] across different studies and has been variable across time.[21] The importance of understanding the prevalence, causes, and geographical distribution of VH is critical to develop and implement tailored interventions to address this public health problem. In this review, we identified the available evidence on COVID-19 prevalence VH in Mexico, with the intent to inform programs and policies aiming to increase vaccine acceptance in the country.

## Material and methods

**Fig. 1:**
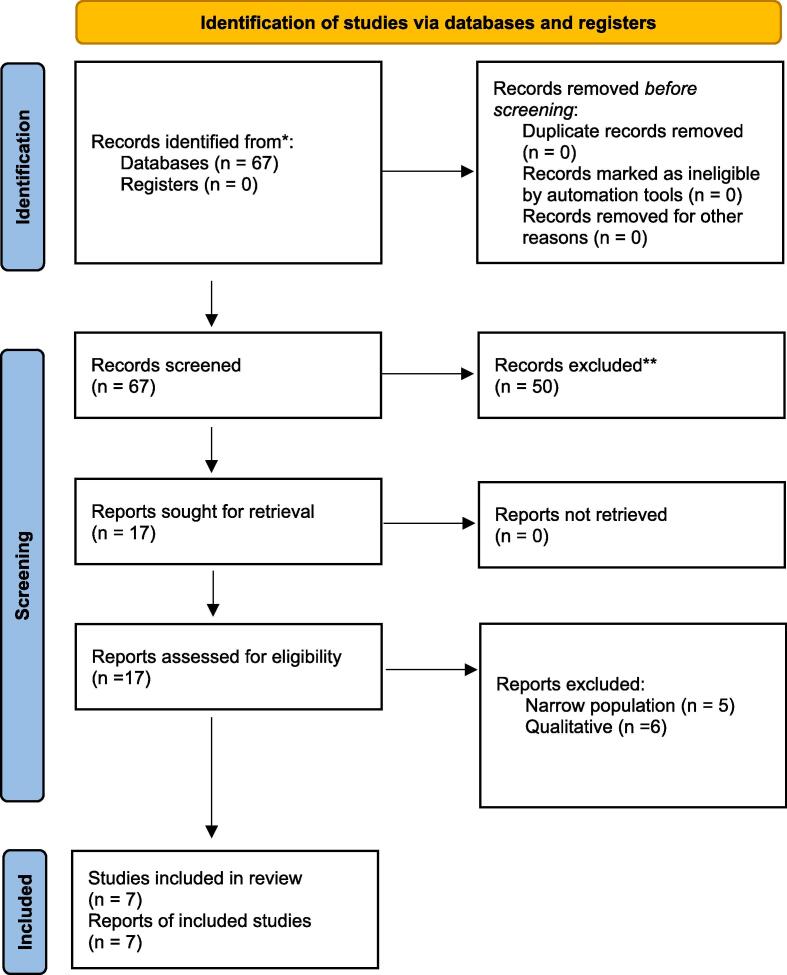
PRISMA 2020 flow diagram for studies included in the analysis.

This study was conducted following the Preferred Reporting Items for Systematic Reviews and Metanalyses (PRISMA) guidelines[22] (Fig. 1). We performed a systematic review of studies published between January 2020 and April 2023 on MEDLINE. The search string included “(vaccine hesitancy) AND (Mexico)”. No language restrictions were applied. Peer reviewed studies assessing vaccine hesitancy in the Mexican population or subgroups were extracted, but only those estimating VH in the whole adult Mexican population were included in the analysis. Two screening phases were performed, a title-and-abstract phase, and a full-text phase. We conducted both in a duplicate and independent manner with pairs of reviewers; two reviewers screened for titles, abstracts, and reviewed full texts. Discrepancies were solved by a third reviewer with experience on systematic review and metanalysis. All records identified by our search strategy were exported to Microsoft Excel software and duplicate manuscripts were removed. We extracted data related to the author, publication year, location, study design, target population, sample size, reported VH point prevalence, survey questions, method of contact and administration, data collection dates, sample size, and risk factors.

The prevalence of COVID-19 vaccine acceptance was reported as a pooled estimate with a 95 % confidence interval. In addition, a sensitivity analysis excluding an outlier study[18] was conducted. A narrative review of studies assessing VH in specific populations was performed. Any study assessing VH prevalence or risk factors among Mexican subpopulations was analyzed. The analysis was performed in R version 4.2.2 using the *metafor* package. To estimate the prevalence, we used a binomial-normal model for *meta*-analysis of proportions (i.e., generalized linear mixed model). Heterogeneity was assessed using the I^2^ statistic.[23], [24].

To characterize the factors associated with VH in the population, an assessment of the VH determinants established by the SAGE working group,[13] including (1) contextual influences, (2) and individual and group influences, was made for the studies included in the pooled analysis. The third construct described by the SAGE group, vaccine/vaccination-specific issues, was not assessed by any of the studies included and is therefore not discussed.

## Results

The initial search strategy identified 67 studies, with 17 full-text articles included for assessed of eligibility. Eleven studies were excluded for narrow target populations not aiming to estimate the VH prevalence of the whole country, or those with a qualitative study design. Seven studies were included for the review and metanalysis (Fig. 1). The studies included were published in 2021 and involved a total of 152,513 adult respondents across all regions in Mexico. All states of Mexico were represented. Table 1 summarizes study characteristics of studies included in the pooled prevalence analysis and studies including subgroups not included in this pooled analysis.

**Table 1:** Study characteristics and reported hesitancy prevalence.

Author	Survey Dates	Date published	Sample Size	Population	Design	Median age (years; [range])	Female (%)	Vaccine hesitancy (%)
^^^Ramonfaur[15]	From 12/04/2020 to 12/11/2020	10/13/2021	3,768	Mexicans, ≥ 18 years, currently living in Mexico	Electronic/Snowball	30 [23–49]	59.6	14.6
^^^Carnalla[18]	From 9/2020 to 11/2020	9/3/2021	10,796	Mexicans, ≥ 18 years, currently living in Mexico	Face-to-Face	nd [30–39]	57.7	37.7
^^^Delgado-Gallegos[20]	From 12/2020 to 02/2021	11/26/2021	1,512	Mexicans, ≥ 18 years	Electronic/Snowball	nd [35–44]	57.9	12.2
^^^Lazarus[11]*	7/2020	3/13/2021	699	Mexicans, ≥ 18 years	Electronic/Snowball	37 [nd]	52.1	23.8
^^^Urrunga-Pastor[19]*	From 1/15/2021 to 2/15/2021	4/16/2021	133,607	Mexicans, ≥ 18 years	Electronic/Snowball	nd	49.8	11.6
^Echánove-Cuevas	From 06/01/2021 to 07/01/2021	08/17/2022	1,195	Mexicans, ≥ 18 years	Electronic/Snowball	32 [18–75]	59	11.0
^Lazarus[12]	From 25/06/2021 to 30/06/2021	01/07/2022	1,000	Mexicans, ≥ 18 years	Multiple international online panel providers.	nd	50.1	18.8
Villarreal-Garza[28]	From 3/12/2021 to 3/26/2021	6/10/2021	540	Women with breast cancer currently living in Mexico	Electronic/Snowball	49 [23–85]	100	34
Guaracha-Basáñez[38]	From 3/01/2021 to 9/13/2021	12/02/2021	600	Mexican outpatients with a rheumatologic condition, ≥ 18 years	Face-to-Face	46 [35–56]	86.5	35.5
Castaneda-Vasquez[33]	From 10/2020 to 11/2020	3/23/2021	543	Healthcare Personnel of the State of Nuevo Leon, Mexico	Electronic	21 [18–69]	65.0	5.5
Guaracha-Basáñez[39]	From 03/01/2021 to 09/30/2021	04/07/2022	1,439	Mexican outpatients with a rheumatologic condition, ≥ 18 years	Electronic	47.4ª [nd]	85.8	27.8
González-Block[29]	From 12/20/2021 to 12/30/2021	09/192022	360	Community dwellers in Mexico City living with or without comorbidities.	Electronic	44.2 [18–86]	50.5	Living with chronic conditions (15 %) Not living with chronic conditions (7 %)
Martinez-Cannon[27]	From 05/02/2022 to 07/22/2022	03/13/2023	201	Patients with cancer undergoing active cancer treatment in a referral center in Mexico.	Electronic	62 [19–92]	57.2	5
Ramonfaur[30]	From 09/15/2021 to 10/30/2021	05/03/2023	1,475	Mexican pregnant people attending a follow-up appointment or at labor & delivery at a maternity center.	Electronic	nd	100	18
Harvey-Vera[32]	From 06/21/2021 to 09/20/2021	06/09/2022	324	Residents of Tijuana who inject drugs and had not received a COVID-19 vaccine.	Face-to-face interview	43.4 (9.6)ª	74.4	36
Mongua-Rodríguez[34]	05/2021 to 06/2021	10/04/2022	840	Students, faculty, and administrative personnel of the National Autonomous University in Mexico	Online/ Electronic	nd	68.1	4

Dates are expressed as (mm/dd/yyyy) or (mm/yyyy) nd: not disclosed, *Multinational studies, ^^^Studies included in pooled analysis, ª = mean (SD), nd = not determined.

### Vaccine hesitancy prevalence

**Fig. 2:**
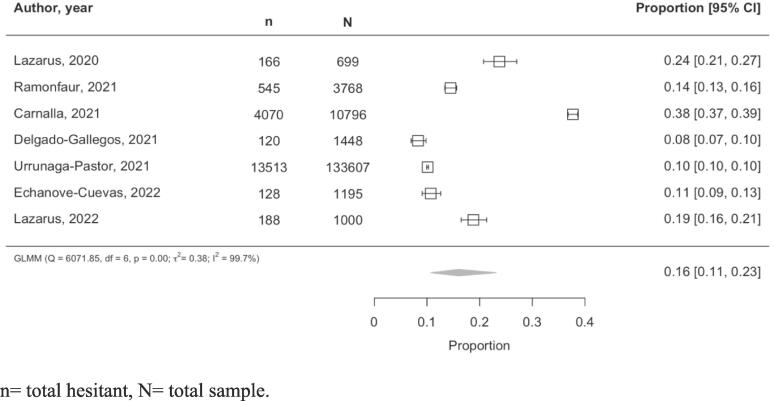
Vaccine hesitancy prevalence pooled estimate.n = total hesitant, N = total sample.

VH was defined as a respondent not being vaccinated against COVID-19 and not being willing to or being undecided about receiving a COVID-19 vaccine,[25] or as defined by each study when a VH binary variable was defined. The point prevalence of VH across included studies ranged from 8 to 38 percent.[18], [20] All studies’ surveys were administered between late 2020 and mid-2021. In the pooled analysis, the point prevalence of vaccine hesitancy in Mexico was estimated at 16 % (95 % CI: 10–24 %; I^2^: 99.7 %; Fig. 2). A sensitivity analysis removing the study with the highest prevalence of VH conducted by Carnalla et al.[18] showed the prevalence to be 14 % (95 % CI: 10–18 %; I^2^: 98.5 %). In said study, VH as defined as “Would you be willing to receive the Covid-19 vaccine once the vaccine is available?”. In total, 28 % of respondents answered “No”, and 9.5 % answered “don’t know” (or did not answer the question).

### Factors affecting vaccine hesitancy

#### Contextual influences

Contextual influence-related factors including age, religion, socioeconomic status (SES), government trust, information sources, and others, were found to be associated with VH in most studies. Of the seven studies included in our analysis, five[12], [15], [18], [20], [26] found that older age and female gender were both strongly associated with VH. Two studies[15], [26] found an association between catholic ideology and VH. Higher SES was associated with higher VH in the study by Ramonfaur et al., and Echánove-Cuevas et al.[15], [26] The study by Carnalla et al. found an inverse association between SES and VH.[18] Ramonfaur et al. and Lazarus et al. found a positive association between education and VH, where those with more education were more likely to be VH.[11], [15] The inverse was found in the study by Carnalla et al.[18] Lazarus et al. and Carnalla et al. evaluated the relationship between residency location and VH.[11], [18] Both studies also found that those that lives in rural areas (compared to urban areas) were more likely to be VH. Ramonfaur et al., Lazarus et al., and Echánove-Cuevas et al. found that low trust in government officials was associated with VH.[11], [15], [26] These studies also found that a higher confidence level toward academic sources as primary sources of information was associated with lower VH. Ramonfaur et al. additionally found that social media or friends and family as primary sources of information was associated with higher VH.[15] Delgado-Gallegos et al. examined the associate between xenophobia and VH, and found a positive association.[20] Further, the latter additionally found that trust in academic sources and evidence-based medicine is negatively associated with the likelihood of VH, whereas low confidence towards the speed of vaccine development and low trust in pharmaceutical companies associated positive with VH.

#### Individual and group influences

Risk perception related to severity of COVID-19, comorbidities, cohabiting with an elderly person, and knowing someone who has died from COVID-19 were associated with lower VH in the study by Ramonfaur et al.[15] This study also found that taking supplements to prevent COVID-19 and having had experienced previous adverse effects of other vaccines were associated with higher VH. In the study by Delgado-Gallegos et al., fear of vaccine adverse events, and low perceived dangers of COVID-19 were associated with lower VH.[20] Lazarus et al., reported that the perception that vaccines can prevent COVID-19, vaccines are safe, and the loss of a family member, associated with less VH.[11] Finally, Echánove-Cuevas et al., reported that participants with VH had a higher number of VH contacts, low perception of COVID-19 risk and high perception of risks related to the vaccine.[26].

### Subpopulations

We performed a narrative assessment of studies evaluating VH of subpopulations in Mexico. Studies assessing VH among people with cancer (n = 2) or other chronic conditions (n = 2), pregnant people (n = 1), healthcare workers (n = 1), people who inject drugs (PWID; n = 1) and within a university community (n = 1) were extracted.

#### Patients with cancer diagnosis

In a study evaluating vaccination status and attitudes towards COVID-19 vaccination among 201 patients undergoing cancer treatment, 95 % of the respondents had received at least one COVID-19 vaccine and 67 % were compliant with WHO guidelines at the time (at least one booster).[27] Nearly a third of the respondents reported “being afraid of side effects” but were not explicitly classified as vaccine hesitant in the study. Moreover, age ≥ 60 years and using mass media as a source of information were highly associated with higher likelihood of having WHO-compliant vaccination status.

In a study among breast cancer patients in Mexico, VH was 37 %.[28] All hesitant patients feared adverse reactions to the vaccine, and had a significantly higher distrust of the healthcare system compared to the non-hesitant participants. Most hesitant women with breast cancer were willing to take the vaccine if recommended by their oncologist. Other individual factors associated with hesitancy in women with breast cancer were younger age (<60 years), education less than high school, not having received an influenza vaccine in the past, and being free of comorbidities.

#### Patients with chronic conditions

González-Block et al. studied VH among those living with a chronic disease including hypertension, gastritis, diabetes, cancer and pulmonary, renal, or cardiovascular disease.[29] Refusal was higher among healthy adults compared to adults with chronic diseases (15 % vs 7 %, respectively). Confidence, complacency, and convenience was similar between groups, healthy adults, and those living with chronic conditions; COVID-19 risk perception was higher among adults with chronic disease.

A multicenter study across three states including 1,439 patients with rheumatologic conditions by Guaracha-Basáñez et al. found a prevalence of VH of 28 %. Hesitant individuals tended to be younger, reported fewer years of education, and had less access to social security benefits. VH was not affected by rheumatoid disease characteristics including diagnosis, disease duration, and comorbidities. There was a tendency toward higher hesitancy among individuals with active disease.

#### Pregnant people

One study among pregnant people in North Mexico found a hesitancy prevalence of 18 %.[30] Among those, over 60 % were willing to receive the vaccine after delivery. Young age and low level of education were associated with higher VH. Catholic religion and more time on social media per day were associated with lower VH. Self-reporting as healthy was associated with higher VH. Participants who had received any other vaccine during pregnancy were 73 % less likely to be vaccine hesitant. Perception of the COVID-19 vaccine potentially affecting the fetus or fertility were strongly associated with higher VH.

#### People who inject drugs

In a study among people who inject drugs (PWID) in the US-Mexico border, those who were born in the USA were more likely to be vaccine hesitant.[31] Those with primary residence in San Diego, CA, were more likely to be vaccine hesitant compared to residents of Tijuana, Mexico. Among Tijuana residents who had not received a COIVD-19 vaccine,[32] 30 % were vaccine hesitant, of whom 40 % later received a COVID-19 vaccine after being referred to a pop-up vaccine clinic.

#### Healthcare workers

Among healthcare workers, COVID-19 VH prevalence was as low as 5.5 %. In this population, misinformation related to vaccination was the strongest predictor of hesitancy, while higher age, being a non-physician, and believing COVID-19 was part of a world-wide conspiracy were also associated with higher VH. The main reasons for not considering vaccination were the possibility of pain or discomfort, potential adverse effects, and little perceived utility.[33].

#### University community members

In a study among university community members, a total of 6 % of the study population reported VH.[34] In this study, VH was associated with younger age, lower education, unpaid employment, having had COVID-19, being free of hypertension, and not having a history of recent influenza vaccination. Hesitant individuals were more likely to have lower health literacy, less trust in physicians, and increased fear related to vaccine adverse effects.

## Discussion

This study provides a pooled estimate of the prevalence of COVID-19 VH (16 % prevalence; 95 % CI: 10–24 %) and explores factors associated with VH in Mexico, among a variety of populations. Our findings suggest that, in Mexico, younger people have higher hesitancy compared to their older counterparts.[11], [15], [18] This is consistent with similar studies in low- and middle-income countries (LMIC) including in Latin America.[35] Risk factors for VH were discordant across studies, reflecting a regional variation in attitudes toward vaccination. In a sensitivity analysis, we excluded the study by Carnalla and collaborators,[18] which reported a disproportionately high prevalence of VH compared to the other six studies. The findings in their study are not solely explained by their definition of VH, where 28 % denied the intention to get vaccinated and 9.5 % were undecided or did not answer the question. The reason this study estimated a much higher prevalence of VH compared to the rest of the studies is uncertain, although having conducted the survey before safety and efficacy of vaccines were known could have contributed. The findings in our analysis were practically unchanged after removing this study, which adds robustness to our results. Overall, this metanalysis provides a valuable estimation of the prevalence of COVID-19 VH in Mexico. Given high consistency in the results and the precision in our estimates, there is a moderate to high level of certainty in our findings.

Vaccine hesitancy is a complex and dynamic phenomenon. As most studies included in our analysis were conducted early during the COVID-19 pandemic, given the dynamic nature of the COVID pandemic, our prevalence estimate is likely very context-specific. Mexico state officials report that 83 % of the adult population had received at least one dose of the vaccine by late 2021. Regardless of the reason why the remaining 17 % had not been vaccinated, this is compatible with the prevalence of VH estimated in this study – 16 %. Our estimate of 16 % VH is more consistent with early attitudes regarding the vaccine, with the study reporting the highest prevalence of hesitancy (38 %)[18] being done before the first data on safety and effectiveness were released.

Our findings suggest the implications of VH are different in vulnerable people with higher risk of severe disease or death from COVID-19. The only study directly comparing VH between healthy adults and those living with comorbidities suggest a significantly higher prevalence of VH among those living with comorbidities.[29] VH in higher-risk populations in Mexico may be higher compared to other Latin American countries, particularly in older adults.[36] In a study among US residents, 18 % of respondents with chronic conditions reported VH.[37] Among respondents with cancer (n = 5,459), 13 % indicated VH, a much lower prevalence compared to the study by Villarreal and collaborators on breast cancer in Mexico,[28] but higher compared to the study by Martinez-Cannon, et al. that reported that 95 % of their patients undergoing cancer treatment at an academic center in Mexico City had received at least one dose.[27] In the same study conducted on US residents with severe diseases,[37] patients with autoimmune conditions (n = 4,946) reported a VH prevalence of 19 %. Surveys conducted in Mexico among patients living with a rheumatologic condition estimate a high prevalence of VH in this population (27 to 36 %).[38], [39] Together, these data suggest that patients in Mexico living with severe chronic conditions may have higher prevalence of hesitancy compered to adults with similar conditions in the US. These findings underscore the importance of conducting vaccine research on vulnerable populations at early stages of epidemics, to quickly release information on the safety and efficacy of vaccines among those who are at higher risk of severe disease.

Studies among pregnant people consistently show much higher VH compared to the general population. In one study, among 5,294 respondents across 16 countries showed 52 % intended to receive COVID-19 vaccine during their pregnancy.[40] This study also shows that countries with economies similar to Mexico’s have lower prevalence of VH among pregnant women. Additionally, a study in the US suggests Hispanic women have higher odds of VH compared to white women for any pregnancy vaccine.[41] In another study conducted among pregnant people in North Mexico in 2021,[30] only 18 % of respondents reported VH. Risks related to the safety of the fetus are the most important drivers of hesitancy in this population. Pregnant people who refuse to get vaccinated should receive follow-up after delivery to not miss the opportunity to get vaccinated to prevent COVID-19 complications.

Among PWID, residents of Tijuana, Mexico have lower VH compared to residents of San Diego, USA, despite there being more vaccine accessibility in the latter.[31] A high proportion of residents of Tijuana were vaccine hesitant (36 %), although, almost half of them accepted to be vaccinated within the 10-month study period.[32] These findings underscore the potential influence of accessible vaccine clinics on the decision to receive the vaccine. In this analysis, no risk factors for VH were found among PWID in Tijuana, which was likely precluded by a low statistical power in the study.

University community members and healthcare personnel have lower rates of VH compared to the Mexican population. These people, who tend to be at less risk for severe COVID-19, have the lowest prevalence of VH. Interestingly, in the study at the UNAM, a highly respected public university in Mexico, the demographic risk factors for VH were similar to those in developed countries, where low age and low education associated with a higher prevalence ratio of VH.[34].

Strategies to increase vaccine uptake considering age, socioeconomic status, and comorbidities must be developed in Mexico. Equitable access to vaccines should be prioritized, as access is a strong predictor for vaccine acceptance, particularly in vulnerable populations.[32] Successful interventions to address VH in Mexico must be tailored to the specific contextual, individual, and group influences. Access to safe and effective vaccines from western countries favors acceptance compared to less effective vaccines from non-Western countries.[15], [42] Horizontal community engagement strategies – collaboration fostering an equal exchange of ideas, resources, and support among all members of a community – will likely result in more successful and equitable vaccination campaigns,[43] without undermining future mass vaccination efforts. Low trust in government is a consistent predictor of VH.[15], [44] While media strategies targeted at reducing VH may not be effective,[43] it is reasonable to emphasize the importance of succinct, consistent, and honest governmental communication about vaccines particularly regarding safety and effectiveness. The role of social media is increasingly being recognized to have a major role in attitudes towards vaccination.[15], [30], [45] The social norms of vaccination are also an important driver of VH. An early study in Puebla, Mexico, suggests susceptibility to COVID-19 and perceived severity affect the perceived social norms, which in turn affect the intention to get vaccinated.[46] However, in this study, the perceived risk of contracting COVID-19 is not associated with intention to be vaccinated.

There are several gaps that remain to better understand VH in Mexico. As the COVID-19 epidemic evolves, new science is required to assess the implications of new vaccine policy issues. Our understanding of VH in Mexico is still limited. Studies exploring VH in young populations and those at lower risk of severe disease are imperative, as the reasons behind VH in these groups may be different compared to those at higher risk of severe disease. Research on how incentives and disincentives impact VH would be useful to tailor interventions. These gaps have scientific interest and political relevance to aid in addressing VH in Mexico.

This study has limitations. Some studies included in our analysis were conducted before vaccine effectiveness for the Pfizer vaccine was publicly released. This likely overestimated VH prevalence. Further, study heterogeneity was very high (I^2^: 99 %); however, this is an expected finding in *meta*-analyses of proportions.[47] Another limitation is that VH is a continuous spectrum and not a binary construct although it is often measured in a binary or categorical scale, introducing residual confounding. This may be problematic because the cutoff for considering someone as hesitant could vary across groups, potentially misclassifying subjects by using a dichotomous definition.

The prevalence of COVID-19 VH in Mexico is 16 %, although this may reflect VH during the early side of the pandemic. Risk factors for VH in Mexico are variable and differ across populations. Characterizing the facets that play a role in accepting the COVID-19 vaccines is crucial for developing strategies to mitigate this public health issue. Further studies on target subpopulations and on ways to address VH are needed to enhance our understanding of this complex phenomenon that undermines public health.

## Search strategy and selection criteria

References for this Review were identified through searches of MEDLINE (via PubMed) with the search terms “(Vaccine hesitancy) AND (Mexico)”. Articles were also identified through a snowball method. Only papers published in English and Spanish were reviewed. The final reference list was generated based on relevance to the scope of this Review.

## CRediT authorship contribution statement

**Diego Ramonfaur:** Conceptualization, Data curation, Formal analysis, Investigation, Methodology, Project administration, Writing – original draft, Writing – review & editing. **Rupali J. Limaye:** Conceptualization, Supervision, Writing – review & editing. **David E. Hinojosa-González:** Conceptualization, Formal analysis, Writing – original draft, Investigation, Methodology. **Francisco J. Barrera:** Conceptualization, Data curation, Formal analysis, Methodology, Writing – original draft, Visualization. **Gloria P. Rodríguez-Gómez:** Conceptualization, Data curation, Project administration, Writing – original draft. **Carlos Castillo-Salgado:** Conceptualization, Project administration, Supervision, Writing – review & editing.

## Declaration of competing interest

The authors declare that they have no known competing financial interests or personal relationships that could have appeared to influence the work reported in this paper.

## Data Availability

Data will be made available on request.
